# Food allergies and allergens in Lebanon: Characterization and perceptions toward labeling

**DOI:** 10.1016/j.waojou.2023.100743

**Published:** 2023-01-20

**Authors:** Berna Elrahi, Zeina Mehanna, Suzan Haidar, Mireille Serhan, Hussein F. Hassan

**Affiliations:** a Department of Family & Consumer Sciences, Sam Houston State University, Huntsville, TX, USA.; b Nutrition Program, Department of Natural Sciences, School of Arts and Sciences, Lebanese American University, 1102 2801, Koraytem, Beirut, Lebanon; c Department of Nutrition and Food Sciences, School of Arts and Sciences, Lebanese International University, Salim Salam, Beirut, Lebanon; d University of Balamand, Faculty of Health Sciences, Department of Nutritional Sciences, Deir El Balamand, Tripoli, Lebanon; e Department of Natural Sciences, Nutrition Program, School of Arts and Sciences, Lebanese American University, 1102 2801, Koraytem, Beirut, Lebanon

**Keywords:** Food allergy, Food allergens, Knowledge, Attitudes, Lebanon

## Abstract

**Background:**

Food allergy is a life-threatening medical condition of public health concern. The aim of our study was to characterize food allergies, in terms of sources, symptoms, severity, and history, as well as to assess the knowledge, practices, and attitudes towards food allergens and allergies, in addition to food allergen labeling, in Lebanon.

**Methods:**

For this, 1100 participants filled over the phone a comprehensive valid questionnaire composed of 41 questions.

**Results:**

Fruits were reported as top food allergens (29.6%), while itching and rash were the most reported symptoms (9.6% and 8.0%, respectively). In terms of knowledge, participants scored on average 67.9 ± 16.2%. Participants who identified as females, below 35 years, highly educated, and from health backgrounds had a significantly higher score (p < 0.05), while area of residence did not have any significant effect (p > 0.05). Participants who are medically diagnosed with allergies and those with health background were found to check the ingredients list and read nutritional claims significantly more frequently than those from a non-health background and who are non-medically diagnosed, respectively, while females were found to check ingredients list and read nutritional claims significantly more frequently (p < 0.05). The majority reported that ingredients are easy to understand (63.2%) and simple to read (61.3%), while allergic individuals agreed that the font size of the ingredients list is not big enough (56.8%) and that E-code numbers are not understood (68.7%). The majority considered it “important” to have the label writings larger and bolded (85.7%), to use simple language (95.5%), to place allergen warning (82.2%), and to use a striking allergen symbol (93.5%).

**Conclusions:**

Our results emphasize the need for national awareness campaigns to improve knowledge and practices, and to lobby policymakers for appropriate management of food allergies and allergens in Lebanon.

## Introduction

A food allergy is a hypersensitivity that occurs when an individual is exposed to a food allergen. This immune reaction can lead to a variety of symptoms such as skin reactions, respiratory issues, and even major anaphylactic shock that can be fatal.^28^ According to Food Allergy Research and Education (FARE), the 9 major food allergens are milk, eggs, wheat, fish, shellfish, peanuts, tree nuts, soy, and sesame.^7^ The National Health and Nutrition Examination Survey estimated that the prevalence of food allergies in the United States was 9%, with 7% and 10% among children and adults, respectively.^26^ In the Middle East, several studies aimed to examine the prevalence of food allergies and identify the most common food allergens among different populations.^2^^,^^3^^,^^9^^,^^19^

The prevalence of food allergies is increasing worldwide. Given the serious health, financial, and nutritional impact this adverse immune reaction has, food allergies have become a critical health care issue.^1^^,^^17^^,^^35^ The main method of managing food allergies is the complete avoidance of the triggering food allergen.^5^ Therefore, the proper labelling of food ingredients and the enforcement of precautionary labelling by governing health authorities is essential for the protection of the diagnosed individuals.^6^ Lebanon requires all food manufacturers to include the list of ingredients in the labelling of the prepackaged food products. Yet, the government does not mandate the presence of precautionary allergy labelling.^22^ Accordingly, an emphasis should be made on the importance of properly educating the public about food allergies, the most common food allergens, as well as the correct methods of managing an allergic reaction in case of its occurrence.^4^

The level of knowledge, beliefs, and practices regarding food allergies among the public has not been widely researched in the Middle East.^9^ The findings of these few studies shed light on the importance of a mandatory allergen warning label. Moreover, they highlighted the necessity to conduct further research regarding the level of understanding of food allergies and allergens among the general population, and not just among diagnosed individuals.^9^

Despite the importance of the aforementioned studies, they either had a small sample size or they included a specific age group. Therefore, no studies have evaluated yet the prevalence of food allergies, in addition to the knowledge, attitudes, and behaviors regarding food allergens in a large sample size with different age groups that are representative of the Lebanese population. Considering the scarcity of data on the topic, further studies need to be performed to acquire an accurate estimation of the prevalence of food allergies in the Lebanese population and to determine the most frequently reported food allergens and associated symptoms. Such studies will allow health authorities to evaluate the magnitude of this issue and act accordingly. Therefore, the aim of this study is to assess the knowledge, attitudes, and practices towards food allergies and allergens among the general Lebanese population, as well as to estimate the prevalence of diagnosed food allergies, the most common food allergens, and associated symptoms. This study will aid in highlighting the importance of proper diagnosis and education concerning this issue, and will help the concerned health authorities in making informed decisions regarding future food allergy-related policies.

## Methods

### Study population

The study recruited a convenient sample of 1100 adult individuals from Lebanon whose ages ranged between 18 and 65. This number was proportionally distributed and stratified into the 5 administrative regions (Beirut, Mount Lebanon including Beirut suburbs, North, South, and Bekaa) (Table 1). Due to the social distancing restrictions enforced due to the COVID-19 pandemic, trained research assistants collected data via phone between March and November 2020. They explained the objectives of the study, ensured the anonymity and confidentiality, and obtained consent from the participants. The interview duration was approximately 20 min. The completion of the questionnaire was voluntary and anonymous, and no incentive was given. This study was approved by the institutional Review Board at our university. Incomplete or inappropriately filled questionnaires were excluded (n = 9) and eventually the final sample size included in the analyses was 1091 (99.2%).

**Table 1:** Characteristics of the sample (N = 1091)

Demographics	N	%
**Gender**		
Male	530	48.6
Female	561	51.4
**Age**		
18–24	666	61.0
25–34	207	19.0
35–44	77	7.1
45–54	61	5.6
55–64	52	4.8
65 and above	28	2.6
**Education level**		
Below High School Certificate	43	3.9
High School Certificate	90	8.2
Technical Degree	57	5.2
Bachelors	769	70.5
Postgraduate studies/MSc/PhD	132	12.1
**Educational Background (2 categories)**		
Health related	252	23.1
Non-health related	839	76.9
**Area of residence**		
Beirut	248	22.7
Mount Lebanon	376	34.5
South	193	17.7
North	165	15.1
Bekaa	109	10.0
**Do you or does any family member (partner, parent, sibling, cousin) or friend have food allergies?**		
No	811	74.3
Yes	280	25.7
**Medically Diagnosed vs Not**		
No	172	61.4
Yes	108	38.5
**Number of Allergies**		
0	811	74.3
1	213	19.5
2	54	4.9
3	11	1
4	2	0.2

### Questionnaire

The questionnaire used in the study aimed to assess the prevalence, knowledge, perceptions, and attitudes of food allergies and allergens among Lebanese individuals. In addition, food allergy symptoms were characterized. This questionnaire was developed in both English and Arabic and was based on valid questionnaires used in previous studies.^9^^,^^33^ Minor modifications related to language and wording were made to some questions in terms of cultural specificity. The questionnaire was piloted to assess its clarity and the average time needed for its completion.

The questionnaire is comprised of 3 main sections: (1) background information, (2) food allergies and allergen knowledge, and (3) food allergies and allergens attitudes and practices. The first section of the survey which includes 24 questions tackles the general demographic information such as age, gender, educational level, and major of study. This section also includes questions about whether the individual or a member of family suffers from a food allergy, if it is medically diagnosed or not, and to specify the food allergen and its symptoms. This information was self-reported and was not verified through medical records. The second section which includes 12 questions assessed the participant's knowledge of food allergies and allergens. It contains a series of true or false questions regarding the major misconceptions about food allergies. The last section includes five questions and tackles the attitudes and practices of individuals regarding food labelling, ingredients, and nutritional claims. This third section also highlights the possible food labelling issues that the Lebanese population in general and the allergic individuals, in particular, may face as well as suggestions for improvements that can be made.

### Statistical analysis

The Statistical Package for Social Sciences (SPSS) V22 (IBM Corporation, Armonk, NY) was used to perform the analyses and statistical significance was set at a p value < 0.05. Analyzed data were summarized using descriptive analyses. Categorical variables were reported as frequencies and percentages. Continuous variables were reported as mean ± standard deviation (SD). Independent t-tests or chi-square were used to compare continuous or categorical variables, respectively, between those who are diagnosed with food allergies and those who are not. Other comparisons based on different variables (age, gender, education level, medical diagnosis vs not) were also performed.

## Results

### Characteristics of the general population

The study included 1091 questionnaires with the majority being females (51.4%). The majority of the participants were aged between 18 and 34 (80.0%), 70.5% had completed a bachelor's degree and 23.1% had a health-related educational background (Table 1). Regarding allergy-related medical history, 25.7% of the cohort answered yes to either having food allergies themselves or having an immediate family member (partner, parent, sibling, cousin) or a friend who has food allergies. Of those 25.7%, 38.5% are medically diagnosed with a food allergy and 61.4% are not medically diagnosed (Table 1).

### Characteristics of food allergies and allergens among the participants

The most common food allergens reported were fruits (29.6%), spices (21.4%), vegetables (12.9%), and tree nuts (10%), as presented in Table 2. The most common allergic symptoms were skin symptoms (16.5%) mainly itching, followed by mouth and throat symptoms (9.5%), with the most common being swelling of the lips or tongue, and lastly gastrointestinal symptoms (9.3%), with the most prevalent being abdominal pain (Table 2).

**Table 2:** Reported food allergens and symptoms.

Reported food allergens			
Food product	N	% overall (n = 1091)	% of those who have allergies (n = 280)
Fruits^a^	83	7.6	29.6
Spices	60	5.5	21.4
Vegetables	36	3.3	12.9
Tree nuts	28	2.6	10
Fish	21	1.9	8.2
Shellfish	21	1.9	7.5
Wheat (gluten)	21	1.9	7.5
Milk	18	1.6	6.4
Peanut	13	1.2	4.6
Egg	12	1.1	4.3
Soy	2	0.2	0.7
Beverages	2	0.2	0.7
Sesame seeds	0	0	0
Mustard	0	0	0
Other Allergies	43	3.9	15.4

**Reported Symptoms among Allergic Individuals (N** = **280)**		
**Symptoms**	**N**	**%**
**Skin symptoms**	**182**	**16.5**
Itching	106	9.6
Rash	88	8.0
Hive	77	7.0
Swelling	54	4.9
Other skin symptoms	1	0.1
**Mouth/throat symptoms**	**105**	**9.5**
Itchy mouth	24	2.2
Lip/Tongue swelling	45	4.1
Throat tightening	19	1.7
Mouth or throat tingling	15	1.4
Difficulty swallowing	27	2.5
Hoarse voice	17	1.5
Other mouth symptoms	5	0.5
**Breathing symptoms**	**74**	**6.7**
Trouble breathing	43	3.9
Chest tightening	34	3.1
Wheezing	10	0.9
Repetitive cough	20	1.8
Nasal congestion	13	1.2
**Gastrointestinal symptoms**	**102**	**9.3**
Abdominal pain	64	5.8
Diarrhea	30	2.7
Cramps	24	2.2
Nausea	33	3.0
Vomiting	26	2.4
Other GI symptoms	2	0.2
**Cardiovascular/heart symptoms**	**35**	**3.2**
Fainting dizziness	15	1.4
Low BP	7	0.6
Rapid HR	19	1.7
Chest pain	3	0.3
**Other symptoms**	**35**	**3.2**
Anxiety	11	1.0
Headache	20	1.8
Other symptoms	4	0.4

a There could be a possible bias when it comes to fruits since fruits cause more oral allergy syndrome rather than food allergy symptoms

### Knowledge, attitudes, and practices related to food allergies and allergens

The study questionnaire included 19 questions and aimed at assessing the knowledge, practices, and attitudes towards food allergies and allergens. The knowledge score of the participants was 67.87 ± 16.28% (mean ± standard deviation) (Table 3). Almost all participants (89.8%) answered correctly when asked what to do when a person begins to experience allergic symptoms, while only 44.2% of the participants knew that food allergens are usually proteins. Furthermore, almost half of the participants (54.1%) thought that removing the allergen after being in contact with the food is safe and that lactose intolerance and milk allergy are the same conditions.

**Table 3:** Responses and mean score of food allergies and allergens knowledge questions

Question	Correct Answer (*T: true/F; false*)	Correct	
		N	%
People with food allergies can safely consume a small amount of that food	F	806	73.9
Cooking, for example frying, stops food from causing allergies	F	882	80.8
Removing an allergen from a meal, e.g. removing the nuts, after cooking makes the meal safe	F	590	54.1
A food allergy is an abnormal response of the immune system to an ordinarily harmless food or ingredient in a food	T	936	85.8
A food allergy is not common but can be fatal	T	792	72.6
Children may outgrow some food allergies	T	837	76.7
Adults can outgrow their food allergies, especially if they stop consuming that food for a period of time	F	701	64.3
People with allergies come mostly from families in which allergies are common	F	511	46.8
Food allergens are usually proteins	T	482	44.2
Lactose intolerance and milk allergy are the same condition	F	590	54.1
Hidden food allergens are one of the most common causes of food allergy occurrences	T	795	72.9
Persons of a known food allergy who begin experiencing allergic symptoms while or after eating a food should initiate treatment immediately, and go to a nearby emergency room if symptoms progress	T	980	89.8
Adults almost rarely outgrow their food allergy	T	725	66.5
**Mean Knowledge score over 100 (Mean** ± **SD)**	**67.9 ± 16.2**		

When stratified by gender, females had significantly higher food allergies/allergens knowledge scores than males (p-value <0.05) (Table 4). In addition, those aged between 18 and 34, those with a bachelor's degree, and those who come from a health-related educational background had significantly (p-value <0.05) higher knowledge scores (Table 4). On the other hand, those who reported having a food allergy themselves, a family member, or a friend with food allergies did have a slightly higher knowledge score than those who do not, but the difference between their scores was not statistically significant (Table 6). Similar results were observed when we compared the scores based on the medical diagnosis or not for the food allergy, whereby those who were medically diagnosed had a non-significant higher score than those who are not medically diagnosed.

**Table 4:** Relations between characteristics of participants and food allergies/allergens knowledge scores

Independent variable	Knowledge score (%)	p-value
	(Mean ± SD)	
**Gender**		
Male	66.4 ± 16.1	0.004
Female	69.2 ± 16.4	
**Age groups**		
18–34 years	68.5 ± 16.1	0.019
≥ 35 years	65.6 ± 16.9	
**Education level**		
Below high school certificate	62.3 ± 17.8	0.006
High school	63.9 ± 16.5	
Technical degree	66.9 ± 18.7	
Bachelors	68 .3 ± 16.1	
Postgraduate studies/MSc/PhD	70.4 ± 14.6	
**Educational background**		
Health/Sciences	77.4 ± 14.5	0.001
Others	65.0 ± 15.7	
**Do you or does any family member (partner, parent, sibling, cousin) or friend have food allergies?**		
No	67.7 ± 16.8	0.595
Yes	68.3 ± 14.8

**Table 6:** Relations between characteristics of participants and food labelling practices

	Educational background			Degree level		
	Health/Sciences	Others	p	High school or below	Post high school	p
**How frequently do you check the ingredients list for any food allergens upon purchasing and/or before consuming any product?**						
Never	76 (30.2%)	353 (42.1%)	**0.001**	47 (35.3%)	382 (39.9%)	**0.684**
Sometimes	100 (39.7%)	302 (36%)		50 (37.65%)	352 (36.7%)	
All the time	57 (22.6%)	117 (13.9%)		23 (17.3%)	151 (15.8%)	
**How frequently do you read the nutritional claims (statement) for any food allergens upon purchasing and/or before consuming any product?**						
Never	93 (36.9%)	342 (40.8%)	**0.002**	48 (36.1%)	387 (40.4%)	0.65
Sometimes	85 (33.7%)	298 (35.5%)		53 (39.8%)	330 (34.4%)	
All the time	54 (21.4%)	102 (12.2%)		19 (14.3%)	137 (14.3%)	
**Which part of the food labelling should be checked by the food allergic customers?**						
Ingredients	25 (9.95)	101 (12.0%)	0.432	21 (15.8%)	105 (11.0%)	**0.002**
Precautionary information only	18 (7.1%)	81 (9.7%)		17 (12.8%)	82 (8.6%)	
Both ingredients and precautionary information	194 (77.0%)	605 (72.15)		80 (60.2%)	719 (75.1%)	

In order to assess the overall attitudes and practices of the participants towards food allergies, 6 questions were asked addressing food allergy related nutritional claims, ingredients, and food labels. Our findings showed that females tend to check the ingredients and nutritional claims for food allergens significantly more than men (p-value <0.01). In addition, a significantly higher percentage of females (p -value: 0.018) believed that allergic individuals should check both the ingredients and the precautionary material written on the food label before purchasing compared to males (Table 5). Those aged 18 to 34 were found to check nutritional claims related to food allergies more frequently than other age groups (p-value = 0.021) as seen in Table 5.

**Table 5:** Relations between characteristics of participants and food labelling practices

	Gender			Age		
	Male	Female	p	18–34 years	≥35 years	p
**How frequently do you check the ingredients list for any food allergens upon purchasing and/or before consuming any product?**						
Never	229 (43.2%)	200 (35.7%)	**0.008**	341 (39.1%)	88 (40.4%)	0.348
Sometimes	187 (35.3%)	215 (38.3%)		332 (38%)	70 (32.1%)	
All the time	68 (12.8%)	106 (18.9%)		134 (15.3%)	40 (18.3%)	
**How frequently do you read the nutritional claims (statement) for any food allergens upon purchasing and/or before consuming any product?**						
Never	235 (44.3%)	200 (35.7%)	**<0.001**	344 (39.4%)	91 (41.7%)	**0.021**
Sometimes	187 (35.3%)	196 (34.9%)		321 (36.8%)	62 (28.4%)	
All the time	53 (10%)	103 (18.45)		125 (14.3%)	31 (14.2%)	
**Which part of the food labelling should be checked by the food allergic customers?**						
Ingredients	68 (12.8%)	58 (10.3%)	**0.018**	105 (12%)	21 (9.6%)	0.749
Precautionary information only	55 (10.4%)	44 (7.8%)		78 (8.9%)	21 (9.6%)	
Both ingredients and precautionary information	366 (69.1%)	433 (77.2%)		638 (73.1%)	161 (73.9%)	

We also observed that education and field of study affect the participants' attitudes and practices regarding food allergies. In fact, more participants with a higher level of education (Bachelor's degree and above) significantly answered that allergic individuals should check both the ingredients and the precautionary material written on the food label before purchasing compared to those without a post high school education (p-value = 0.002). Moreover, participants with a health-related educational background tend to check ingredients and nutritional claims for food allergens significantly more than those who come from a non-health related educational background (p-values = 0.001 and 0.002, respectively) (Table 6).

The attitudes of those who were medically diagnosed with food allergies were significantly better than those who were self-diagnosed. In fact, medically diagnosed participants tend to check food allergen related ingredients and nutritional claims significantly more than those who are self-diagnosed (p-value = 0.009 and p-value = 0.007, respectively). Moreover, when asked which part of the food labelling should be checked by the food allergic customers, 78.7% of medically diagnosed participants answered both ingredients and precautionary information as compared to 72.7% of self-diagnosed participants, and these results were significantly different (p-value = 0.012) as shown in Table 7.

**Table 7:** Relations between characteristics of participants and food labelling practices

	Medically diagnosed?		
	Yes	No	p
**How frequently do you check the ingredients list for any food allergens upon purchasing and/or before consuming any product?**			
Never	20 (18.5%)	37 (21.5%)	**0.009**
Sometimes	45 (41.7%)	95 (55.2%)	
All the time	38 (35.2%)	30 (17.4%)	
**How frequently do you read the nutritional claims (statement) for any food allergens upon purchasing and/or before consuming any product?**			
Never	24 (22.2%)	56 (32.6%)	**0.007**
Sometimes	39 (36.1%)	76 (44.2%)	
All the time	33 (30.6%)	25 (14.5%)	
**Which part of the food labelling should be checked by the food allergic customers?**			
Ingredients	16 (14.8%)	26 (15.1%)	**0.012**
Precautionary information only	0 (0%)	15 (8.7%)	
Both ingredients and precautionary information	85 (78.7%)	125 (72.7%)	

To further examine participants’ attitudes and practices towards allergen-related food labelling, the questionnaire included 3 questions that revolved around the different aspects of the food label such as ingredients, allergy warning, E-code etc. and the improvements that could be done. As presented in Table 8, the majority of the participants agreed that the ingredient labels printed on food products were easy to understand (63.2%), simple to read (61.3%), and contained enough details about ingredients (48.0%). However, 51.8% of participants agreed that the information provided regarding allergens was not enough for the food allergic consumer.

**Table 8:** Attitudes towards ingredient labels, towards problems encountered when identifying foods for allergic individuals, and towards improvements to labeling

Attitudes towards ingredient labels	Agree	Neutral	Disagree
	%	%	%
*Ingredients: Easy to Understand*	63.2	11.5	25.3
*Ingredients: Simple to Read*	61.3	10.9	27.8
*Ingredients: Enough Details*	48.0	16.7	35.3
*Ingredients: Enough Information*	28.1	20.1	51.8
**Possible problems encountered when identifying foods suitable for a person suffering from food allergy**	**Agree**	**Neutral**	**Disagree**
	**%**	**%**	**%**
Language of the ingredient list and nutritional claim/statement is not easily understood	37.5	22.4	40.1
Placement of the allergy warning or ingredients list on the package is not convenient to read	44.8	23.3	31.9
It is not easy to understand the source of certain ingredients so that I can identify if those ingredients are safe to consume	55.8	21.2	23.0
E-code numbers of additives in the ingredient lists are not understood by me	68.7	16.9	14.5
I cannot find the manufacturer's contact details easily	30.0	28.3	41.6
Font size of the ingredient list and nutritional claim/statement is not big enough to read clearly	56.8	21.4	21.8
**Rate, in your opinion, the level of importance of improvements that should be taken when purchasing food products for a person suffering from food allergy**	**Not Important**	**Neutral**	**Important**
	**%**	**%**	**%**
Writings on ingredients label should be in bold and larger font	7.9	6.4	85.7
Use of simple language (simple Arabic, English or French)	1.9	2.6	95.5
Use of a striking symbol to indicate the presence of allergens for illiterate people	1.6	4.9	93.5
The placement of allergy warnings next to ingredients list	5.9	11.9	82.2
Manufacturer's contact detail	17.3	15.6	67.0

Participants were also asked to assess possible problems a food allergic customer may encounter when identifying suitable food, and their answers are presented in Table 8. A greater percentage of the participants believed that the manufacturer details were clearly stated on the food label (41.6%), while 40.1% disagreed that the language used was easily understood. Nevertheless, the majority believed that placement of the allergy warning needs to be changed and emphasized (44.8%), found difficulty in understanding the source of the ingredients (55.8%) and the E-code numbers of the additives in the ingredients list (68.7%). On the other hand, 56.8% believed that the font size used in the labels should be bigger (Table 8).

Lastly, the participants were asked to rate the level of importance of improvements that should be made when purchasing food products for the food allergic consumer (Table 8). The results showed that 85.7% of the participants believed that the writings on food labels should be in bold and in a larger font. Furthermore, almost all participants (95%) agreed that a simpler language should be used. The participants also agreed that the use of a striking symbol to indicate the presence of an allergen is important to ease the process for the food allergic customer (93.5%). In addition, 82.7% of participants believed that an allergy warning should be placed next to the ingredients list, and 67% of them indicated that improvements should be made in emphasizing the contact details of the manufacturer.

## Discussion

The present study examined the knowledge, practices, and attitudes towards food allergens and allergies, in addition to food allergen labeling, in Lebanon. We observed that these variables varied based on the gender, level of education and field of study of the participants.

With regards to the larger percentage of female participants compared to males in the present study, the literature reported an increased engagement of females compared to males in food-related studies, due to females taking a larger percentage of the responsibility for the household's shopping and meal preparation.^33^ Another reason is that there are gender specific differences in food intolerances, whereby women have been shown to suffer more often from food related symptoms due to hormonal differences, intake of medications, etc.^24^ As for the common food allergens, our results are similar to that of Irani et al,^18^ who reported fruits, nuts, and spices to be among the top 5 most prevalent food allergies among self-reported food allergic individuals in Lebanon, and to that of Sakakini et al,^30^ who reported fruits, vegetables, and nuts to be among the most common allergens among Lebanese school children. Regarding food allergy symptoms, similar results were found in studies conducted in Mauritius and Lebanon, which also classified cutaneous and gastrointestinal symptoms to be the most frequently reported food allergy symptoms.^30^^,^^33^

The knowledge score of the participants (67.87 ± 16.28%), in general, is considered low and shows that more work is needed about raising awareness about food allergies. More action is required from health authorities to increase awareness and knowledge among the general population. Females scored significantly higher than males for the knowledge of food allergies and allergens in our study. Similar results were observed in a study conducted in Saudi Arabia where female schoolteachers had significantly higher knowledge scores than male teachers.^14^ In addition, those aged between 18 and 34, those with a bachelor's degree, and those who come from a health-related educational background had significantly (p-value <0.05) higher knowledge scores. This is in agreement with results of other studies, which stated that having a higher level of education was associated with a greater knowledge of food allergies and food labelling.^11^^,^^25^

In our study, females tend to check the ingredients and nutritional claims for food allergens significantly more than males (p-value: 0.08 and p-value <0.01 respectively). Also, females believed that allergic individuals should check both the ingredients and the precautionary material written on the food label before purchasing compared to males (Table 5). These results are in line with a Jordanian study where females had better attitudes towards food allergies than males.^11^ This is due to females having higher levels of empathy and being more invested in nutritional issues than males.^25^ Those aged 18 to 34 were found to check nutritional claims related to food allergies more frequently than other age groups (p-value = 0.021) as seen in Table 5. This might be that the younger population is more interested in knowing nutrition info and more self-conscious about they eat.

Our findings regarding the effects of education and field of study on the participants’ attitudes and practices regarding food allergies are in line with other studies conducted in the United States and Germany that aimed to assess attitudes towards food allergies. It was observed that higher levels of education were correlated with more positive attitudes towards food allergies and allergens.^25^^,^^29^

The better attitudes of those who were medically diagnosed with food allergies were reported in Ju et al^21^ and Lee et al,^23^ which indicated that individuals with a medically-diagnosed allergy have more positive attitudes towards food allergy related nutritional claims, ingredients, and food labels than self-reported allergic participants. On the other hand, our finding regarding the inadequacy of the information provided about allergens for the food allergic consumer was in line with Cornelisse-Vermaat et al,^8^ who conducted a study in Netherlands and Greece with allergic individuals and found the food allergen related information on the food labels to be insufficient and unsatisfactory. Nevertheless, including additional information on a food label may not be feasible due to space limitation,^34^ and may be overwhelming to the consumer.^31^

Our results about the lack of clarity of the food label language, the font size of labels, and the difficulty in understanding source and e-code numbers of ingredients are in agreement with other studies in the literature. For instance, a study in the United States found that self-reported food allergic individuals believed that the ingredient list used technical words that were not easily understood, and this posed a serious obstacle in managing a food allergy.^36^ Nevertheless, food labeling cannot be applied to fruits as fruits and vegetables are exempt from food labels. Therefore, increased awareness about fruit allergens is needed as this is one of the most common reported food allergies. Furthermore, according to a study conducted in Mauritius in 2018, E-code numbers should be further emphasized on the food label as they provide essential information on the additives and the types of ingredients.^33^ As for the font size, the participants of the studies conducted by^8^^,^^16^^,^^32^ also believed that the smaller font size used on food labels tend to be problematic for the food allergic consumer when identifying suitable food options.

As for the needed improvements to food labels, the majority believed that the writings should be in bold and in a larger font. Similar results were found by Cornelisse-Vermaat et al, Hassan et al, and Ju et al^8^^,^^15^^,^^21^ whose studies found readability of the food label to be problematic. These studies' participants reported that the fonts of the labels should be larger and bolded to increase clarity and readability. This is supported by Mfueni et al^27^ who reported that most food labels tend to use bold fonts when stating the presence of an allergen. Furthermore, the majority of the participants agreed that a simpler language should be used. This is an integral aspect of preventing accidental exposure to the allergen, as a large percentage of participants in studies conducted in different countries (Mauritius, Netherlands, and United States) agreed that the use of complex terminology posed to be an obstacle for food allergic individuals when choosing suitable foods.^8^^,^^20^^,^^33^ The participants also agreed that the use of a striking symbol to indicate the presence of an allergen is important to ease the process for the food allergic customer (93.5%). Similar results were reported by Hassan et al and Soogali et al,^15^^,^^33^ where 97.1% and 87.6% of the participants, respectively, believed that the use of a striking symbol was needed to indicate the presence of an allergen. In addition, 82.7% of our participants believed that an allergy warning should be placed next to the ingredients list. Currently, there are no clear international guidelines on how and where an allergen information should be expressed on the food label.^8^ An asterisk (∗) is commonly used to indicate the inclusion of an allergen in the ingredients list, and the use of the Crossed Grain symbol is used in European countries for gluten free products to indicate that they abide by the proper standards.^13^ Moreover, in 2005, the European Union labelling legislation allowed producers to use terms such as “may contain traces of” or “made in factories where allergens are produced”.^12^ A study conducted by Cornelisse-Vermaat et al^8^ found that using these terms caused participants to become overly restrictive in what they can and cannot eat whereas a similar study conducted by DunnGalvin et al^10^ stated that the term “not suitable for” was preferred to other terms such as “may contain”. These studies indicate the need to set international guidelines for proper labelling of allergen containing food products to prevent accidental exposure and ensure the safety of the allergic consumer. In addition, 67% of our participants indicated that improvements should be made in emphasizing the contact details of the manufacturer. This was also reported in the study of Soogali et al,^33^ where 60% of the participants indicated that the manufacturer's details must be visible. This is a crucial section of the food label as Joshi et al^20^ found that 46% of parents contact manufactures to determine if the allergen is present in the product.

## Conclusion

In conclusion, this study provided insights into the most prevalent food allergies and food allergy symptoms, and assessed the knowledge, attitudes, and practices of the general population towards food allergens and food labels in Lebanon. Strengths of our study include the large sample size that was distributed proportionally to different districts, allowing to have a representative sample of the Lebanese population, increasing the generalizability of our results. On the other hand, our study has few limitations. Because of COVID-19 pandemic restrictions, we had to conduct data collection via phone, and the majority of the information was self-reported. This is noteworthy for the diagnosis of food allergy and its symptoms as over reporting might have ensued especially that we observed a high percentage of non-medical food allergy diagnosis. This might be due to low knowledge about this topic (as evidenced in our results) and to the confusion of other symptoms with signs of food allergy. Therefore, our results need to be interpreted cautiously. Lastly, the medium used to conduct our survey, ie, phone interview, might have affected the outreach to different groups of the population with an easier access to the younger population leading to having the majority of our participants young. This might affect the extrapolation of our results to the older population.

The participants had a generally average knowledge score, identified several possible problems with food labels, and gave their opinions on improvements that can be made, such as using larger fonts, simpler language, clear allergy warnings, and emphasizing manufacturers' details. This finding calls for action to enhance the knowledge and increase awareness about food allergies, keeping in mind that the majority of the sample is young and this score can be skewed by the younger population. The results of this study are of extreme importance to health authorities, policy makers, and food manufactures to ensure the safety of the public and the consumers. Accurate labelling of the ingredients and the allergen is of monumental importance to the food allergic consumer considering that the only treatment of food allergies is the complete avoidance of the allergen, which is greatly dependent on the consumers' ability to comprehend the food label with ease. These findings emphasized the importance of implementing a national food allergy labelling guideline, which includes a mandatory allergy warning. In fact, having a more precise regulation and harmonization of the food allergen labeling, along with a qualitative and quantitative detection methods, will help enhance the consumer's quality of life. That is why a close collaboration among international organizations, food industries, and policy makers should exist towards global implementation of mandatory requirements for allergen labelling more coherently, in addition to harmonized laboratory assays and reference materials.^37^ In addition, these findings highlight the necessity of increasing awareness about food allergies in the Lebanese population through national awareness campaigns. Further studies revolving around food allergy labelling knowledge, attitudes, and practices of caregivers and food handlers must be carried out in Lebanon to identify the improvements and changes needed to be done to protect the food allergic consumer.

## Funding source

Not applicable.

## Availability of data and materials

Available upon request from the corresponding author.

## Author contributions

BE and ZM co-designed the questionnaire, conducted the statistical analysis and co-wrote the manuscript; SH and MS collected the data and co-wrote the manuscript; HH conceptualized the project, co-designed the questionnaire, secured the funding and co-wrote the manuscript.

## Ethics approval

Approval of the study was granted by the Institutional Review Board at the Lebanese American University. IRB approval number: LAU.SAS.HH1.4/Dec./2019.

## Consent for publication

Approved.

## Declaration of competing interest

All of authors report no competing interests or financial disclosure.
